# Effectiveness of bilastine and fexofenadine among allergic Rhinitis patients in Ranchi, Jharkhand, India

**DOI:** 10.6026/97320630018596

**Published:** 2022-06-30

**Authors:** Abha Kumari, Subodh Kumar Paswan, Binod Kumar Sinha, Ritesh Kumar Sinha, Sandeep Kumar

**Affiliations:** 1 Rajendra Institute of Medical Sciences, Ranchi, Jharkhand, India

**Keywords:** Allergic rhinitis, antihistamine, bilastine, fexofenadine

## Abstract

Allergic rhinitis is a worldwide health problem which impairs quality of life and interferes with daily activities. Untreated allergic rhinitis also carries a significant financial burden for the society. Bilastine, a novel second-generation antihistaminic drug that is highly selective for the H1 histamine receptor, has a rapid onset and prolonged duration of action. Thus, the aim of our study was to compare the effectiveness of bilastine and fexofenadine in treatment of allergic Rhinitis patients. 104 patients were enrolled who have fulfilled the inclusion criteria for the study from the OPD of Ear, Nose and Throat Department. Patients were divided randomly in two groups A and B. Patients of group A were allowed to take tab Bilastine 20 mg OD whereas, patients of group B were allowed to take tab. Fexofenadine Hydrochloride 120 mg OD orally for two weeks. The baseline Total Nasal Symptom Score (TNSS) were compared between two groups. The study findings showed that the mean TNSS was significantly reduced in our study group. Baseline TNSS was 13.55 and 13.45 in Group A and Group B respectively. Reduction in this parameter first become apparent in the 24 hours and maintained till 2nd week. Bilastine showed significant improvement in quality of life of Allergic rhinitis patients and proved to be more effective than fexofenadine in reducing the TNSS score, when used alone in allergic rhinitis patient.

## Background:

Allergic rhinitis is a symptomatic condition of nose caused by allergen exposure of IgE-mediated inflammation [1]. The characteristic manifestations of allergic rhinitis are sneezing, Rhinorrhoea and nasal obstruction. In addition, patient with allergic rhinitis can experience troublesome non-nasal symptom, such as headache, thirst and difficulty in sleeping, as well as cough, wheezing, sinus pressure, sore throat and ocular symptom such as itchy, redeye or epiphora [2]. Allergic rhinitis is a worldwide health problem. Prevalence of allergic rhinitis is estimated to be 10-30% [3]. This disorder is likely to affect the lives of >500 million people worldwide [4]. This burden is particularly more where people used to live in crowded places with high level of environmental pollution [5]. Allergic rhinitis impairs quality of life and interferes with daily activities.Untreated allergic rhinitis also carries a significant financial burden for the society in terms of costs of medication, physician visits, hospitalizations, and loss of productivity at work. Thus, effective treatment of allergic rhinitis is imperative [5]. Allergic rhinitis is classified as "intermittent" (symptoms present for <4 days a week and <4 consecutive weeks), "persistent" (symptoms present for > 4 days a week and for more than 4 consecutive weeks), "mild" and "moderate-severe" according to the allergic rhinitis and its impact on Asthma (ARIA) guidelines [6]. In moderate-severe types of allergic rhinitis one or more of the symptoms such as sleep disturbances, impairment of daily activities, sports, leisure, impairment of school or work and troublesome symptoms are present. H1 histamine receptors are involved in allergic reactions [6]. Degranulation following entry of an allergen to immunoglobulin E (IgE)‑sensitizes mast cells that causes the release of histamine which is responsible for the symptoms of allergic rhinitis.Since decades, first generation antihistaminic drugs have been used in the management of allergic rhinitis, but because of lipophilic in nature it readily crosses blood brain barrier leading to central nervous system side effects such as sedation, and drowsiness that affects the routine life of the society. Apart from that being short half-lives of first-generation antihistaminic drugs it requires multiple daily dosages. Hence, newer antihistaminic drugs were introduced to minimise the side effects and multiple daily dosing of 1st generation drugs to improve the compliance of the patients. Second generation antihistamines are lipophobic, having low capacity to cross blood brain barrier, thus reducing sedation and cognitive impairment. They have lower affinity for non-histamine receptors and higher specificity for binding to H1 receptors. These drugs have longer half-lives, allowing once or twice daily dosing [6]. As Fexofenadine, an active metabolite of terfenadine is the second generation antihistaminic drug is a nonsedating, selective histaminic H1 receptor antagonist having rapid and long-acting activity with no anticholinergic effect [7]. Bilastine is a newer, well tolerated, nonsedating H1 receptor inverse agonist having a high specific affinity for H1 receptor that possesses both antihistaminic as well as anti-inflammatory properties in vitro as well as in vivo [8, 9]. Moreover, it has rapid onset of action and longer duration of effect. Bilastine is a substrate for P-glycoprotein, an organic anion transporting protein (OATP) that prevents its uptake across the blood brain barrier into the brain [10]. It has no impact on CYP450 enzyme of liver. It does not have any drug interaction, except that there is an increased uptake of Bilastine if taken simultaneously with diltiazem, erythromycin or ketoconazole [11]. It is depicted from preclinical in vitro study that Bilastine has shown to dose-dependently inhibit binding of H1 receptors in the guinea pig cerebellum, with an affinity approxi-mately threefold greater than that of cetirizine and fivefold greater than that of Fexofenadine [12]. Although, Fexofenadine is most commonly prescribed in ENT OPD to treat allergic rhinitis patients, but Bilastine being higher affinity for H1 receptor in guinea pig cerebellum with safe and long duration of action, a comparative study in terms of effectiveness in patients of allergic rhinitis has been conducted. Therefore, it is of interest to compare the effectiveness of Bilastine and fexofenadine in treatment of Allergic Rhinitis patients.

## Materials and methods:

This was an observational, single centred, two arm, parallel-group, comparative clinical study conducted in the Department of pharmacology & Therapeutics, among the patients attending out patient department of Ear, Nose and Throat (ENT) Department. Before starting the study, approval from Institutional ethics committee was taken (199, REC, RIMSdt.21-12-2019). Informed Consent was taken from all patients and Case Report Forms were maintained separately for each patient. Total 104 patients were enrolled who have full filled the inclusion criteria. For each patient, the duration of scheduled treatment was 2 weeks and the total duration of study and analysis was 16 months.

## Study methodology:

Patients were divided randomly in two groups A and B. Patients of group A were allowed to take tab Bilastine 20 mg OD whereas, patients of group B were allowed to take tab. Fexofenadine Hydrochloride 120 mg OD orally for two weeks.

## Evaluation of total nasal symptom score:

Total nasal symptom score (TNSS) was composed of the sum of five individual symptom scores (sneezing, rhinorrhoea, nasal obstruction, nasal itching and difficult sleep), each symptom assessed every 15 min on a 4-point scale: 0 = none (no signs/ symptoms), 1 = mild (symptom clearly present but easily tolerated), 2 = moderate (symptom bothersome but tolerable), 3 = severe (symptom difficult to tolerate – interferes with activities). The maximum score for TNSS was 15 for each recorded time point.

Following procedure will be performed on the day of the participant enrolment. After collecting written informed consent from all the study patients, enquiry about the medical history was performed. Then, physical examination and vital sign was recorded. The preformed questionnaires of TNSS recording Sheet were explained to every study participant. Then, each and every patient was asked to fill the preformed questionnaires of TNSS recording sheet at the interval of 24 hours, 7th day and 15th day. After Completion of study on 15th day, the completely filled TNSS recording Sheet were collected and then data collected was transferred to master chart. Statistical analysis was performed by using Statistical Package for the Social Sciences, IBM SPSS 20. The data was tabulated as mean ± standard deviation (Mean ±SD). Paired 't' test was used to compare mean changes in TNSS Score before and after treatment. P-value < 0.05 was considered statistically significant.

## Results:

Flowchart of the present study is given in Figure 1(see PDF). For efficacy mean changes in TNSS at the end of 24 hr, 1st and 2nd weeks were evaluated. 47% of males and 53% of females belonged to group A. 43% of males and 57% of females belonged to group B. (Figure 2 - see PDF) 39.90+ 7.71Paired t test showed statistically significant difference between group A and group B (Table 1 -see PDF). Figure 3(see PDF) represents mean changes in TNSS from day 0 to after 1 day, 1 week and 2 weeks in both group A and B. TNSS scores decreased at 2 weeks.

## Discussion:

The current study was carried out by Department of Pharmacology along with Department of ENT to observe the comparative efficacy evaluation of Tab Bilastine and Fexofenadine in patients of moderate to severe case of Allergic Rhinitis. Out of 104 patients, only 102 patients completed the study according to the protocol. One patient was lost to follow-up at the end of the 1st week in Bilastine group (Group A) and one patient failed to complete 2weeks in Fexofenadine group (Group B) and was not integrated in the analysis (Figure 1 -see PDF). The two groups were homogeneous with respect to baseline demographic data. The demographic characteristics of two groups were compared for age and sex. In our study, female is predominance in both groups. This result is similar of meta-analysis done by Frohlich et al. which shown that prevalence of coexisting allergic rhinitis with asthma and those with allergic rhinitis, females were predominant [13]. The baseline Total Nasal Symptom Score (TNSS) were compared between two groups. The mean TNSS was significantly reduced in our study group. Baseline TNSS was 13.55 and 13.45 in Group A and Group B respectively. Reduction in this parameter first become apparent in the 24 hours and was maintained till 2nd week. The mean change of TNSS score was 8.71 in Group A and 9.78 in Group B from baseline to 24 hours. Bilastine was been found better than Fexofenadine in decreasing TNSS in allergic rhinitis patient from baseline to 24 hours. Similar order of change in TNSS was observed in study by Horak Friedrich et al. which showed that Bilastine and cetirizine were both significantly more effective than Fexofenadine [14]. Similarly, the mean change of TNSS score was 6.94 in Group A and 7.65 in Group B from baseline to 1st week. Bilastine have been found better than Fexofenadine in decreasing TNSS in allergic rhinitis patient from baseline to 1st week. A study was observed by Kuna P et al. was in agreement with our study findings where the percentage decreases TNSS was significantly greater with Bilastine and cetirizine than placebo [15]. The mean change of TNSS score was 2.7 in Group A and 3.43 in Group B from baseline to 2nd week. Bilastine was found to be better than Fexofenadine in decreasing TNSS in allergic rhinitis patient from baseline to 2nd week. Another study by Bachert et al. proved thatBilastine 20 mg significantly reduced the TNSS from baseline as compared to Desloratadine and placebo. [16] Similar results were observed in both groups, but the reduction with respect to this parameter was greater in Group A as compared to Group B. A similar order of changes in TNSS was observed in our study after 1 week and 2 weeks of treatment, thus, Bilastine was found to be superior to Fexofenadine in decreasing TNSS in allergic rhinitis patient.

## Limitations of study:

The study could have been performed in large number of patients; a larger sample size gives better results. It can be conducted for longer duration, so that symptoms disappear completely and outcomes of study would be reliable. Brand change of study drugs can lead to error in outcome values.

## Conclusion:

In this study, the drugs showed significant improvement in quality of life of Allergic rhinitis patients. Bilastine had shown more effectiveness compared to fexofenadine in reducing the TNSS score, when used alone in allergic rhinitis patients.

## Future research:

There is very limited data regarding effectiveness and safety of Bilastine in pregnant and lactating women. Thus, more studies should be conducted. There is a need to assess and analyse the pharmacokinetic, efficacy and safety of bilastine in pediatrics population in liquid dosages form for ease of convenience.
